# Supplementary material for: Are carbon emissions trading and green financial instruments synergistic? -Comprehensive quantitative research based on content analysis
Source: PLoS One. 2024 Mar 7;19(3):e0298601. doi: 10.1371/journal.pone.0298601 (PMC10919601; doi:10.1371/journal.pone.0298601)
Supplement: S1 Data — (ZIP) [file pone.0298601.s001.zip › Data/地级市Z_F工作报告.zip!/╕╩╦α/╞╜┴╣╩╨/└┤╫╘╣1⁄2╓┌║┼í╛┬φ┐╦╩2╛▌═°í┐_2017.docx]

各位代表：

现在，我代表市人民政府，向大会报告工作，请予审议，并请政协委员和列席人员提出意见。

过去五年工作回顾

市三届人大一次会议以来的五年，是平凉发展史上很不平凡的五年。五年来，面对复杂多变的宏观经济形势和艰巨繁重的改革发展任务，在市委的坚强领导和市人大、市政协的监督支持下，我们全面贯彻党的十八大和十八届三中、四中、五中、六中全会精神，深入学习习近平总书记系列重要讲话，认真落实“四个全面”战略布局和五大发展理念，紧紧围绕市三次党代会确定的思路目标，牢牢把握稳中求进总基调，统筹推进稳增长、调结构、促改革、惠民生、防风险各项工作，较好地完成了市三届人大一次会议确定的目标任务，全市经济健康发展，民生持续改善，社会和谐稳定，迈出了转型升级、科学发展的新步伐，为全面建成小康社会奠定了坚实基础。

五年来，我们主动适应经济发展新常态，坚持不懈抓投资、促消费，经济实力稳步提升。全面落实“3341”项目工程，以市列“十大工程”为引领，共实施500万元以上项目6258项，完成投资2374亿元，年均增长12%。认真落实促进消费的各项政策措施，积极搭建消费平台，建成了陇东农副产品批发交易中心、平凉国际商贸城、平凉工业园区综合物流中心、金果博览城等一批商贸物流设施，线上线下互动的商贸流通服务网络日趋健全，电商平台、家政服务、健康养老等新型业态应势而生，城乡消费日趋活跃，社会消费品零售总额达到196亿元，年均增长13.4%。预计到年底，全市地区生产总值达到381亿元，五年净增105亿元，年均增长9.3%；公共财政预算收入达到24.97亿元，财政支出突破170亿元，分别是2011年的1.6倍和1.7倍；固定资产投资跨上了600亿元台阶，较2011年翻了一番多，对经济增长的贡献率达到37%；城乡居民人均可支配收入分别达到23640元和7282元，较2011年增长75%和103%。

五年来，我们聚焦全面建成小康社会，坚持定向施策、精准发力，脱贫攻坚取得阶段性成效。突出“六个精准”，融合推进双联行动和“1236”扶贫攻坚行动，细化落实 “1+17”“2+19”精准扶贫举措，富民产业培育、基础设施改善、公共服务保障、能力素质提升取得明显成效。粮食产量连续五年保持在100万吨以上，果品产量、蔬菜产量、肉牛出栏量分别较2011年增长54.4%、52.5%和28.6%，以现代农业“五个百万”增收工程为重点的特色产业收益占到农村居民收入的一半以上。强化劳动力技能培训和有组织输转，年输转劳务45万人，人均创劳务收入15800元。整合各类资金，易地搬迁贫困群众1.3万户5.9万人，完成建制村通畅工程4890公里，解决了56.65万农村人口饮水不安全问题和13.9万人饮水不稳定问题，改造农村危房11.8万户，农村居民彻底告别了土窑洞和地坑庄子，贫困村标准化卫生室、幼儿园覆盖率分别达到70.8%和61.6%，动力电实现了全覆盖。有效破解扶贫资金短缺难题，探索建立了扶贫贷款使用监管、村级互助资金筹措使用等制度。预计到年底，可实现脱贫59.8万人，贫困面由41.5%下降到10.5%。

五年来，我们着眼构建现代产业体系，坚持扩量提质、优化结构，产业转型升级迈出坚实步伐。加快转变农业发展方式，引进建办了秦宝牧业、海升果业、正大饲料、方盛蔬菜等一批农业产业化龙头企业，组建农民合作社856家，发展家庭农场1100个，以新型农业经营主体为引领的现代农业加快发展。坚持煤电产业优化升级与壮大非煤产业同步推进，《陇东能源基地开发规划》《灵台矿区总体规划》获得国家批复，陇东特高压直流输电工程已列入国家“十三五”能源规划及电力专项规划待国务院审批，平凉电厂二期和60万吨煤制甲醇建成投产，邵寨、五举、赤城等一批矿井项目稳步推进，20万吨聚丙烯项目加快建设；10万锭棉纱、5万吨木浆纸一期、欣叶纸箱精包装、庆华陶瓷等项目形成了新的产能，光伏晶硅切片、碳纤维隔热材料、生物胶原蛋白肽等新兴产业项目相继建成，中德合作立体停车库生产基地加快建设，非煤工业占规上工业增加值比重由2011年的16.8%提高到28.5%，煤电主导、多元发展的工业化格局正在形成。集中实施华夏文明传承创新区“163”计划，以构建崆峒山、大云寺·王母宫等大景区为基点，实施崆峒山度假山庄、美年文化城等文旅融合项目236个，新增文化企业543家，“神奇崆峒·养生平凉”文化旅游品牌影响力进一步扩大，旅游接待人数、综合收入分别是2011年的3倍和3.4倍，以大旅游为引领、大文化融合为特色、大商贸服务为配套的第三产业快速发展，增加值年均增长12% 。

五年来，我们围绕破解体制机制障碍，坚持改革创新、开放开发，发展活力进一步释放。大力推进简政放权，建成市县公共资源交易平台、三级政务服务中心和“三张清单一张网”，市本级行政审批事项减少86.2%，非行政许可审批事项全部取消，商事制度改革全面推行，非公经济实体净增4.2万户，增长87.6%，增加值比重提高到45%。财税金融改革走在全省前列，“营改增”试点全面推开，国库集中支付和公务卡应用改革全面推行，化解政府逾期债务11.9亿元，盘活预算单位结转结余资金5.5亿元。设立市长金融奖，组建了惠民、泓源、富民等投融资实体企业，发行企业债券15亿元，实现融资218.2亿元。甘肃银行、交通银行、银河证券等6家金融机构在平设立分支机构，新增金融便民服务网点2354个，全市贷款余额达到540亿元，较2011年翻了一番多。持续深化农村综合改革，集体林权制度改革在全国创造了经验，农村土地承包经营权确权登记颁证有序推进，规范流转土地93.25万亩。不动产登记、国有林场、供销社等改革取得积极进展。主动融入丝绸之路经济带建设，成功举办了一系列重大节会赛事，多层次、多形式、多领域招商富有成效，中电建、华信、杭萧钢构、苏宁云商、国电南瑞等一批全国知名企业落户平凉。

五年来，我们立足全面改善基础条件，坚持统筹兼顾、产城融合，城乡面貌发生显著变化。西平、天平铁路和西长凤高速建成通车，全市等级公路总里程增加了一倍多，实现了县县通二级公路、乡乡通油路、村村通硬化路。引洮二期静宁骨干工程开工建设，实施了一批高标准基本农田、流域治理、节水灌溉工程，基本建成了“自来水化市” “梯田化市”。新建330千伏、110千伏变电站16座。实施3G网络升级改造、4G网络新建、广电网络数字化双向升级改造等信息基础设施项目67项，电信“宽带乡村”示范工程顺利推进，光纤宽带传输网络覆盖城乡。坚持规划引领，全市编制各类规划795项，中心城市率先在全省建起了覆盖地上地下、层级完备的规划体系，五年累计投入基础设施建设资金62亿元，相继建成了一批骨干道路、重要节点、城市景观、重大市政工程，形成了“六横十二纵”路网框架，主城区实现了集中供热、雨污管网全覆盖，生活垃圾无害化全处理。6县县城和小城镇建设取得明显成效，抓建市列城乡一体化试点乡镇21个，建成美丽乡村51个、新农村示范村506个、环境整洁村851个，撤乡改镇28个，全市建制镇达到60个，城镇化率提高到38.97%。坚持绿色发展，加强生态建设和环境保护，五年完成植树造林115.6万亩，治理水土流失1186.4平方公里，新修梯田103万亩，森林覆盖率、城市绿地率分别提高到31.1％和34.6%。集中开展了大气污染防治联合攻坚，实施重点节能减排技改项目328项，淘汰落后产能90万吨，空气质量优良天数年均达到290天，单位生产总值能耗、主要污染物排放量控制在目标值之内。

五年来，我们致力提升人民生活水平，坚持构建体系、兜底保障，群众获得感不断增强。认真落实政策，强化资金配套，养老、医保、低保、就业、住房等社会保障体系全面建立，保障范围不断扩大，保障水平逐年提高。基本养老保险实现全覆盖，城乡居民、企业退休职工养老金较2011年分别增长54.5%和75.6%，五年发放110.5亿元，年受益人群达到34.2万人，机关事业单位养老保险制度改革启动实施。城镇居民医疗保险、新农合政府补助标准均由200元提高到420元，累计为128.7万人（次）报销住院费用41.5亿元。城乡低保标准分别增长87%和160%，年均保障28万人，累计发放保障资金26.36亿元。就业形势保持稳定，在全省率先开展高校毕业生“支企”服务，多途径安置高校毕业生3.7万人，累计新增城镇就业18.7万人，城镇登记失业率控制在4%以内。实施各类保障性住房和城市棚户区改造4.4万套，城镇人均住房面积达到30平方米，城镇低收入住房困难家庭实现了应保尽保。

五年来，我们着眼促进公共服务均等化，坚持共建共享、惠民利民，各项社会事业取得长足发展。坚持每年承诺措办一批惠民实事，财政用于民生的支出累计达到565.9亿元，是前一个五年的2.2倍，在总支出中的占比达到77.7%。新建、改建校舍265.2万平方米，乡镇中心幼儿园实现全覆盖，学前教育入学率提高到74%，义务教育均衡发展水平在全省领先，平凉职业技术学院建成招生，平凉医专升格为甘肃医学院，填补了我市本科院校空白。改扩建市妇幼保健院、市级全科医师培养基地等市县级医疗机构38个、乡镇卫生院79个，新建标准化村卫生室681个，每千人拥有床位数实现了翻番，县级公立医院综合改革全面推开，在全省率先开展了分级诊疗试点。全市文化“三馆一站”实现免费开放，市广电新闻中心、体育健身广场建成投用，市博物馆建成主体，建成乡村舞台1219个、广播电视“村村通”2306处、“户户通”28.8万户，县区数字影院、“农家书屋”实现全覆盖，文化惠民、卫生健民能力不断增强。持续深化普法教育，平安平凉建设深入推进，一些影响社会稳定的突出矛盾和信访积案得到有效化解，食品药品安全监管不断细化深化，安全生产形势稳定向好。宗教事务依法管理水平稳步提高，“两个共同”示范区建设成效显著。

五年来，我们着力加强政府自身建设，大力转变政府职能，从严从实改进作风。自觉接受人大法律监督、工作监督和政协民主监督，坚持重大事项向人大报告、与政协协商制度。认真听取各民主党派、工商联以及无党派人士意见建议。办理人大代表建议417件、政协委员提案557件。组建了市政府法律专家咨询委员会，健全了重大行政决策公众参与、专家论证、风险评估、合法性审查和集体决定制度。深入开展党的群众路线教育实践活动、“三严三实”专题教育、“两学一做”学习教育，严格执行中央、省市委关于改进作风相关规定，全市“三公”经费支出逐年压减。认真落实党风廉政建设“3783”主体责任，加强对公务人员特别是领导干部的经常性教育和监督管理，强化审计监督和行政监察，突出公共资源交易、项目招标、政府采购等重点领域监管，严肃查处发生在群众身边的“四风”和腐败问题，政府系统廉政建设不断加强。统计调查、档案、外事、侨务、地震、气象、人防、地方志、红十字、老龄、残疾人事业取得新的成效，双拥共建、国防动员、民兵预备役等工作深入开展。

各位代表，过去的五年，是抢抓机遇、应对挑战、开拓奋进的五年，是综合实力稳步提升、城乡面貌显著变化、人民生活水平明显提高的五年。在经济下行压力加大、发展基础薄弱、自然灾害多发的情况下，我们能够取得这些发展成绩，得益于省委、省政府和市委的坚强领导，得益于市人大、市政协的监督支持，得益于全市广大干部群众的艰苦奋斗。在此，我代表市人民政府，向全市各族人民，向各民主党派、工商联、无党派人士和人民团体，向中省驻平单位，向驻平部队和武警官兵，向所有关心支持平凉发展的各界人士，致以崇高的敬意和衷心的感谢！

各位代表，五年的艰辛实践使我们深刻体会到，要打赢脱贫攻坚战，全面建成小康社会，必须坚持发展第一要务不动摇，对认准的目标，一张蓝图绘到底，一届接着一届干，凝心聚力，攻坚克难，在不断强产业、促转型、夯基础、增实力中走出符合平凉实际的发展路子；必须坚持项目建设不松劲，始终强化项目抓手，主动适应投资导向，超前谋划，全力争取、引进和实施一批大项目、好项目，依靠项目调整优化结构、厚植发展优势、改变城乡面貌、支撑经济社会发展；必须坚持改革创新不止步，着力破除体制机制障碍，全面创优发展环境、创业环境，提升宜居宜业聚集效应，在开放开发中不断激发经济社会发展的内生动力；必须坚持改善民生不懈怠，不折不扣落实惠民政策，努力扩大公共服务覆盖面，着力解决人民群众最关心最直接最现实的利益问题，不断提高人民群众的幸福感；必须坚持改进作风不停顿，认真践行“三严三实”，强化责任担当，严格考核奖惩，以一流的政风、行风和作风，保障和促进经济社会稳定健康发展。

各位代表，在看到成绩的同时，我们也清醒地认识到，经济社会发展中还存在着不少困难和问题，主要表现在：经济总量小、人均占比水平低，支撑发展的大项目、产业项目尤其是工业项目少，经济增长后劲不足；未脱贫人口相对较多且贫困程度深，自身发展能力不强，脱贫攻坚难度大；产业链条短、结构不尽合理，农业产业化程度还不高，工业经济高度依赖煤电的格局还未彻底改变，新兴产业培育缓慢，现代服务业发展层次低，转型升级任务艰巨；创新能力不足，经济、管理、科技人才短缺，投融资渠道单一，民间资本还未完全激活，资金瓶颈制约仍然比较突出，新经济业态还处在萌芽阶段；基础设施建设相对滞后，机场、高铁等快速通道建设推进缓慢；资源约束趋紧，节能降耗、治污减排、环境保护的压力依然很大；城乡居民收入水平偏低，就业渠道不宽，持续增收的压力加大；教育、文化、医疗等公共产品供给不足，还不能很好地满足人民群众需求；少数干部责任意识、担当意识不强，抓落实的力度不大，懒政、怠政、不作为的问题在一些单位仍然程度不同地存在，工作能力和水平还不能适应形势发展的要求。对此，我们将采取更加有力的措施，认真研究解决。

未来五年发展思路目标和2017年工作任务

刚刚闭幕的市第四次党代会，科学规划了未来五年建设幸福活力美好平凉的宏伟蓝图，这是全面建成小康社会的现实选择，是广大人民群众的共同愿望，也是政府工作的根本遵循。未来五年政府工作的总体要求是：高举中国特色社会主义伟大旗帜，以邓小平理论、“三个代表”重要思想、科学发展观为指导，全面贯彻党的十八大和十八届三中、四中、五中、六中全会精神，深入贯彻习近平总书记系列重要讲话精神，全面落实“五位一体”总体布局和“四个全面”战略布局，积极适应经济发展新常态，深入贯彻五大发展理念，奋力决胜全面小康，以打造全省经济转型升级先行区、循环经济示范区、“一带一路”重要节点城市、商贸物流中心和全国文化旅游养生地、生态文明建设示范市为战略支撑，坚守发展、生态、民生三条底线，更加注重推进改革创新，更加注重转型升级发展，更加注重补齐小康短板，更加注重生态文明建设，加快建设幸福活力美好平凉。

新起点、新目标、新任务，对政府工作提出了新的更高要求。我们将认真落实市第四次党代会精神，以贯彻五大发展理念为总要求，以决战决胜全面小康为总任务，深入实施“十三五”规划，按照全力实施“八大战略”、努力实现“八个提升”的具体部署，集中各方智慧，凝聚全社会力量，突出“八个着力点”，精准发力，集中突破，协调推进，切实把建设幸福活力美好平凉的各项战略举措落到实处。着力补短板。认真贯彻习近平总书记全面小康不能有一个贫困家庭、一个贫困群众掉队的重要指示精神，把片区化攻坚与插花户脱贫统筹起来，全面落实精准扶贫政策举措，大力提升劳动力素质，全方位拓展产业扶贫、旅游扶贫、电商扶贫、光伏扶贫以及双联行动等有效模式和做法，进一步深化脱贫攻坚的帮扶内涵和考核内涵，全力打好富民产业发展、基础设施建设、公共服务提升“三大战役”，前三年集中攻坚，后两年巩固提高和冲刺扫尾，确保新标准下所有贫困人口脱贫，村出列、县摘帽，与全国全省一道步入小康社会。着力破瓶颈。下功夫解决资金、交通两大制约。适应新的投融资政策，做大做强现有融资实体，对接各类资本运作模式，盘活国有资产，以符合市场需求、国家投资导向为前提，以功能互补、资产优良为基点，每个县区和工业园区（集中区）至少建办起一个融资平台，采取市场化运作模式设立政府投资基金。强化资金规范运作，撬动民间资本、社会资本参与项目建设，促使一大批基础设施、工业经济、产业升级的好项目，按照新的资本运作模式快速落地达标，发挥应有的效益。深入推进“6873”交通突破行动，加快实施平庆、宝中二线、西平二线等铁路项目，建成平凉机场和平天、彭大等7条高速，升级改造15条国省道，构建市通高铁航运、县县通高速、乡乡通国省道的立体化综合交通网络，全面提升交通通达能力。着力创环境。坚持生态立市，实施生态绿化五年行动计划，完成植树造林260万亩，建设一批公园、林荫大道、生态廊道，全面加大环境治理力度，加快建成国家级生态文明建设示范市、国家森林城市，打造山清水秀自然美的生态环境。加快法治政府、责任政府和服务型政府建设，持续提升各级政府服务发展的能力，着力形成人才、资本、技术的洼地效应，以优良的软环境促进软实力快速提升。着力抓项目。突出项目主引擎作用，强化项目储备，设立重大项目可研基金，建立分产业、分行业的项目论证机制，足额预算项目前期论证经费，大中小并举，一二三产涵盖，可研高度深度达标，促使向上争取符合要求、对外招商互利多赢。重点围绕现代农业快速提升、煤电化冶转型升级、文旅商贸提质扩量等方面，谋划论证实施一批上亿元、十亿元、百亿元的大项目、好项目，打造一批大企业和企业集团，形成产业集群优势，以项目建设的大突破推动全市经济快速发展。着力强支撑。以科技创新为引领，加快打造特色产业升级版，突出抓好重点矿区、重大煤电化冶以及电力外送通道、油气资源开发等项目，加快建设千亿级煤电化冶循环经济产业集群；以农业产业化龙头企业、农民合作社、家庭农场为载体，全面推动农村特色优势产业和新兴产业提质增效、助农增收；快速融入“一带一路”，充分发挥区位优势，建设一批专业市场、商贸中心、物流集散中心，加快建成陕甘宁交汇区重要物流基地；以打造大景区为引领，推进文旅融合，建设一批旅游专业村镇，打造市域精品旅游环线和通达周边、辐射全国的旅游圈，以大景区带动大旅游。深入实施工业强市战略，以新能源、新材料、新装备、新技术、新产品为重点，持续做大做强光伏、风电、碳纤维、生物质能等战略性新兴产业，打造新的工业增长极。以新机制、新模式、新服务全面推动工业园区（集中区）扩容升级，平凉工业园区跻身国家级经济技术开发区，4个工业集中区升级为省级工业园区。着力增活力。持续推进重点领域和关键环节改革创新，加大简政放权力度，全面落实投资项目报建审批清单制度，提升服务项目、服务企业、服务基层的效率，不断激发市场主体竞相发展的活力；认真落实支持创新创业政策，搭建一批低成本、便利化、全要素、开放式、接地气、接市场的创业平台，不断激发创业创新活力；支持各级干部担当干事、有为干事、激情干事，为全市经济发展增动力、添活力。着力惠民生。突出普惠共享，全面落实国省各项保障政策，持续加大民生投入，建立以普惠性政策为主、各级政府配套措施跟进的保障体系，每年措办一批惠民实事，下功夫解决上学难、就医难等群众关注关心的热点难点问题，努力让群众享有更稳定的劳动就业、更可靠的社会保障、更优质的文化教育、更高水平的医疗保健、更舒适的居住条件。着力提实力。通过五年的不懈努力，确保2021年地区生产总值突破550亿元，人均达到2.4万元以上，公共财政收入达到33亿元，城乡居民收入增速高于经济增速，综合实力在全省位次前移，加快建成经济强、百姓富、保障好的幸福平凉，市场主体多元、产业配套协同、创业热情高涨的活力平凉，山清水秀、宜居宜业、文明和谐的美好平凉。

2017年，是全面贯彻落实市第四次党代会精神的起步之年，是新一届政府全面履职的开局之年。全市经济社会发展的主要预期目标是：地区生产总值增长7.5%，规模以上工业增加值增长6%，固定资产投资增长10%以上，社会消费品零售总额增长10%，公共财政预算收入同口径增长8%，城镇居民人均可支配收入增长10%，农民人均可支配收入增长12%，城镇登记失业率控制在4%以内，居民消费价格指数控制在103%以内，人口自然增长率控制在9.8‰以内，单位生产总值能耗和主要污染物排放完成省上控制指标。

重点做好八个方面的工作：

（一）突出精准施策，奋力推进脱贫攻坚。推动“2+19”政策措施到村到户到人，确保130个贫困村2.4万户9.6万人脱贫，华亭、泾川、灵台3县摘帽。加快发展富民多元产业。持续突出牛果菜全产业链开发，推进现代农业“五个百万”增收工程提质增效，培育壮大新型农业经营主体，完善与农民利益联结机制，新建标准化养牛小区20个，推广苹果矮化密植园2万亩，加快泾汭河川区国家级蔬菜产业园、海升12万平方米智能温室栽培生态示范项目建设。强化宣传推介，推动平凉金果等优势农产品与大市场有效对接，建立稳定购销关系，实现产业增效、农民增收。积极发展油用牡丹、中药材等区域性特色产业，实现有条件的贫困村、贫困户全覆盖。粮食产量保持在110万吨以上。持续做大劳务经济。完善职业培训、就业服务、劳动维权“三位一体”工作体系，整合各类培训资源，对建档立卡贫困户免费开展订单、订岗、定向精准培训，健全劳务市场对接机制，扩大技能型、资本积累型、人才型劳务输出。进一步打响“庄浪梯田人” “陇原妹”等劳务品牌，提高劳务经济的层次和水平。全年输转劳务47万人，创劳务收入80亿元。加大基础设施改善力度。集中力量改善贫困户住房条件，加快实施危房改造和易地搬迁，所有退出村全部消除D级危房。加快村组道路建设，巩固提升安全饮水成效，新建一批小型农田水利、抗旱应急水源工程，改造110千伏变电站3座、35千伏变电站4座，新建10千伏以下配电线路1570公里，实现村村通光纤。扩大公共服务保障覆盖面。逐户落实义务教育控辍保学措施，健全完善贫困乡村中小学校及幼儿园教师补充、村医全员培训机制，加快标准化村卫生室、幼儿园、文化活动中心等公共服务向贫困村覆盖进程。落实兜底对象应保尽保措施，加大重特大疾病救助和农村低保、五保供养等帮扶力度，坚决兜牢社会保障底线。健全精准帮扶工作机制。对建档立卡贫困村、贫困户、贫困人口定期进行核查调整，按照新的标准，强化动态管理，完善到村到户帮扶机制，落实帮扶措施。健全脱贫攻坚监督考核机制，细化扶贫资金监管办法，确保脱贫攻坚到村到户到人措施落得实、抓得准、能到位、有成效。

（二）突出谋争引建，强力推进项目建设。坚持项目支撑、投资拉动，千方百计抓谋划、抓争取、抓招商、抓落地，完成固定资产投资764亿元以上。开展储备项目大谋划。精心谋划生成一批投资规模大、带动力强、成长性好的重大项目，坚持抓大不放小，做深做细项目可研论证、审核报批、信息对接、资金方案等工作，杜绝“一句话、一张纸”项目，提高项目成熟度，增强项目可行性、可批性和实效性，确保项目库储备规模保持在5000亿元以上，其中工业项目达到一半以上。重点加快平凉机场、定平庆铁路、宝中二线客运专线、陇东特高压直流输电工程、白龙江引水平凉支线工程、崆峒水库改扩建等项目前期工作。开展产业项目大招商。着眼链条延伸、多元互补，对论证成熟的项目，带着可研精准招商，带着国家投资意向融合招商，带着地方支持政策聚焦招商，围绕现代农业经营主体培育、煤电化冶一体化开发、大物流大枢纽打造、旅游大景区要素完善和工业园区（集中区）孵化器、创业园建设等方面，高层次、宽领域、针对性地招大引强，实招商、招实商，确保招商引资签约规模达到600亿元以上，资金到位率增长12%以上。开展重点项目大建设。筛选推出一批PPP、EPC项目，激活民间投资，吸引更多社会资本投入项目建设。集中实施过10亿元项目28项，重点加快平天、彭大两条高速和312线改造、平华一级等4条国省道建设，新建平庆、平泾等5条省级高速和泾源至秦安等4条省道。开建引洮二期静宁段配套工程和庄浪梁河等3座水库，抓好灵台新集水库建设。建成太阳山—六盘山—平凉750千伏输变电工程，配合实施新疆昌吉—安徽古泉1100千伏特高压输变电工程。全年实施500万元以上项目1105个。

（三）突出构建多元格局，促进工业经济稳中有升。坚持传统产业存量提升与新兴产业增量扩张并重，强化协调调度，推动工业经济规模效益双提升。持续推进煤电化冶一体化开发。抢抓国家对贫困地区能源开发的差异化政策机遇，加快实施《陇东能源基地开发规划》《灵台矿区总体规划》，积极争取国、省减量置换政策，复工建设邵寨、五举煤矿，力争唐家河、安家庄煤矿获得核准，启动南川河、泾川高平北、高平南及灵北等矿井项目前期工作。加快发展煤化工产业，力促华煤20万吨聚丙烯建成投产并开展发泡材料、注塑产品、无纺布、塑木复合材料4个下游产品开发项目的前期工作。力争华亭电厂二期核准，平凉电厂三期、崇信电厂二期列入全省火力发电优选项目，加快打造煤电化冶循环经济产业集群。着力培育工业经济新增长点。启动新一轮重大技术改造升级工程，全力实施《中国制造2025平凉行动方案》，推进制造业和互联网融合一体发展。支持杭萧钢构、智能立体停车库生产基地、众一兴旺管材等项目达产达标，力促庆华陶瓷二期、宝马纸业5万吨高速木浆纸、俐源7万吨精细丁基再生胶粉等项目建成投产，加快天纤棉业20万锭棉纱、鑫盛建材二期建设，推动建材、装备制造业转型升级。加快光伏、碳纤维、生物制药、煤机高端装备产业园建设，推进庄浪、静宁、泾川3个集中式光伏电站建设，力争500兆瓦时锂离子动力电池项目开工，加快新能源汽车推广应用，建成一批充电设施，战略性新兴产业增加值增长10%以上。深入推进百户民营小微企业帮扶工程，中小企业户数增长500户以上，增加值增长7.5%以上。大力提升园区配套承载能力。深化平凉工业园区售电侧改革试点，配套完善道路、供水、供电、通讯等基础设施，年内引进过亿元项目10个以上，培育一批年产值过百亿元的骨干企业。加快静宁、华亭工业园区管理体制改革，探索建立企业参与管理、政企合理担责的运营体制，不断增强吸纳力和聚集力。

（四）突出文旅融合发展，不断提升壮大第三产业。强化龙头带动，拓展融合领域，培育发展新业态，第三产业增加值增长10%。推动文化旅游产业上水平。全面实施文明素养提升、旅游品牌打造、文化产业培育、文化服务惠民四大工程，把建设华夏文明传承创新区与打造大旅游经济圈有机融合，深度挖掘景区景点文化内涵，完善景区管理体制，全面推进大景区和中华崆峒养生地建设，加快原生态民俗文化体验区、崆峒山生态旅游度假山庄一期、胭脂峡漂流谷生态农业观光园、关山大景区基础设施提升等重点文旅项目建设，开建自驾游营地4个，力争崆峒山建成全省首批8个大景区之一，大云寺·王母宫创建国家5A级旅游景区取得实质性进展，争取关山景区跻身全省大景区行列。积极发展红色旅游，加快界石铺红军长征纪念园建设，打响界石铺红色品牌。大力发展乡村旅游，实施旅游专业镇、专业村规范达标工程，建成旅游专业村7个、标准化农家乐100户。全年旅游综合收入增长22%。推动商贸物流扩规模。加快平凉南铁路综合货场建设，增强陇东农副产品批发交易中心、平凉工业园区综合物流中心、金果博览城等商贸平台的辐射带动能力，抓好义乌国际商贸城等重点商贸设施建设，新建改建一批便民市场和商贸惠民工程，加快县有配送中心、乡有农贸市场、村有便民市场全覆盖进程。推动新业态快成长。大力发展电子商务，积极推广苏宁云商模式，建立本地化统一结算电商平台，进一步提升县级电子商务中心运营水平，抓好国家级电子商务进农村示范县和省级电子商务示范县、示范园区创建，扶持建办一批乡镇、村社电子商务平台，电子商务交易规模增长20%以上。加快发展互联网金融、股权投资类企业。大力发展生产性、生活性服务业，积极发展医疗保健、健康养生、文体休闲、养老家政等新型消费，满足群众多样化、个性化需求。

（五）突出中心带动，聚力推动城乡一体发展。科学统筹生产、生活、生态空间，以基础设施建设为基点，推动公共服务向农村延伸，加快构建以中心城市为龙头、县城为节点、小城镇为纽带、美丽乡村为支撑的新型城镇体系。强化规划的战略引领和刚性约束。改革完善规划体系，加快中心城市绿地景观规划、海绵城市规划及公共服务、综合交通、幼儿园、停车场等民生规划的编制实施，加强县乡基础设施、公共服务设施专项规划和特色小镇、美丽乡村建设规划修订完善，抓好静宁、华亭两县“多规合一”试点。依法从严查处违规建设行为，深化公众参与机制，建立规划监管数字平台，切实增强规划的科学性、法定性和强制性。持续提升中心城市品质。突出生态宜居，加快城市面山绿化和崆峒大道、泾河两岸林荫景观廊道建设，实施崆峒山景前区湿地公园、虎山公园、龙隐寺公园和7条景观绿带、3处街头游园建设工程；突出以人为本，加快新民北路西片二期、天门塬、太统路等12个片区棚户区改造续建，实施果木寺巷、兴北路等9个片区棚户区改造新建，启动老旧小区综合改造，抓建一批文明示范小区，推进热电联产集中供热二期工程，强化居住小区幼儿园、卫生所、便民市场、公厕、养老中心等公共设施配套；突出基础设施提升，建成泾河大道东段、东环路、北大路，拓建泾河北路，打通新民北路等断头路，完善城市主干路网框架，改造提升给排水、供热供气等老旧管网，维修维护破损道路、路灯等市政设施；突出文化传承，加强历史文化遗迹保护，注重老城修复，彰显城市文化底蕴；突出精细管理，改革城市管理体制，创新城市治理方式，推进智慧城市建设，启动建设平凉市云中心，构建大数据服务平台，提升城市管理的人性化、精细化、智能化水平。统筹推进县城、重点小城镇建设。抓好省列“两县三镇”和市列“八镇”新型城镇化试点，推进六县县城功能完善、基础配套和品质提升，加快排水、污水、集中供热等地下管网建设和污水处理厂扩容改造，实现污水全收集、全处理和集中供热全覆盖，增强城市承载、综合服务和辐射带动功能。强化重点小城镇建设，打造一批历史文化印记深刻、特征明显的特色小镇。加快推进户籍制度改革，强化居民素质教育，引导农村转移人口就近就地城镇化，力争全市城镇化率提高2.7个百分点。集中开展治堵、治污、治脏、治乱行动，提升城镇文明形象。切实加快美丽乡村建设。突出城乡结合部治理，开展“百村美丽”示范工程建设和村庄整洁工程，挖掘村落文化，突出乡村特色，保持田园风貌，因地制宜，随形就势，抓建省级美丽乡村示范村20个、市县级美丽乡村40个、环境整洁村200个。

（六）突出生态建设，持续拓展发展空间。大规模植绿播绿。启动国家森林城市创建，以城市园林化、城郊森林化、乡村林果化、道路林荫化、院落景观化为目标，认真实施天保二期、三北五期、新一轮退耕还林、重点公益林补偿等林业重点生态工程，全民动员、全社会参与，大规模开展义务植树造林，集中抓好公园绿地、滩涂湿地以及城镇街区、水源地绿化，推进机关单位、住宅小区拆墙透绿、破硬增绿，人均公共绿地面积提高到10.77平方米，着力塑造通山显水、绿网交织、满城显绿的人居环境；实施城镇面山治理、破损山体植被恢复工程，建设城乡交通主干道绿色通道96条1300公里；推进景区扩量增绿，加强森林资源保护，创建一批森林公园和湿地公园。全年完成造林绿化50万亩，森林覆盖率提高到31.5%。加强水保生态治理，新修梯田15万亩，治理水土流失330平方公里，治理河堤35公里。深入开展环境污染治理行动。健全完善大气污染防治长效机制，突出扬尘、餐饮油烟、机动车尾气、工业废气污染防治，完成平凉电厂4号、6号机组超低排放改造，推动大气环境质量稳定好转。加强泾河、汭河、达溪河、葫芦河流域地表水和城市黑臭水体治理，推进集中式饮用水源地环境保护和地下水污染防治，切实改善水环境质量。实施农药化肥零增长行动，加强规模化畜禽养殖污染防治。强化重点行业、重点企业环境执法监管，坚决打击环境违法行为。切实加强资源集约节约利用。严守耕地红线，清理闲置土地，加大土地开发利用强度。落实最严格的水资源管理制度，启动中水回收利用，提高污水再生利用率。加大工业固体废物利用，综合利用率达到83.5%。加强工业、建筑、交通和公共机构等重点领域节能，加快企业循环化生产、产业循环化组合、园区循环化改造，实施循环经济及资源综合利用项目20个。

（七）突出改革创新，着力激发发展活力和动力。加快重点领域改革。持续推进“放管服”改革，继续取消下放一批审批事项，不断简化流程、规范程序，加强权责清单运行动态监管，扩大“双随机、一公开”覆盖面，加快推行“网上行权”。全面落实企业“五证合一、一照一码”和个体工商户两证整合登记制度。稳步推进农村综合改革，完成土地承包经营权确权登记颁证，开展农村集体产权股份合作制改革试点，抓好水权、水价、水利建管体制改革，推进国有林场、供销合作社综合改革，扩大政策性农业保险范围。加大政府购买公共服务力度。加强财税金融改革，健全完善财权与支出责任相匹配的运行机制，有效防范和化解政府性债务风险。完善“营改增”后续管理，大力培育地方主体税源。深入推进普惠金融和扶贫金融，强化小额贷款、投资担保等机构监督管理，依法打击非法集资和信用违约行为。分类推进国有企业改革，推动政府部门管理企业改制脱钩。全方位扩大开放开发。依托“一带一路”开放大通道，实施互联互通、产业对接、经贸合作、人文交流、战略平台五大工程，办好第四届平凉金果博览会、崆峒养生文化旅游节、关山大景区全国山地自行车邀请赛等重大节会。扩大与“一带一路”沿线国家和周边地区在商贸物流、文化旅游等领域的合作交流，建立具有区域特色和民族特色的外贸公共服务平台，支持市内骨干企业“走出去”，全市进出口总额增长10%。大力推动大众创业万众创新。认真落实扶持政策，引导社会资金和金融资本融入创业创新活动，大力培育众创、众包、众扶、众筹等新兴业态，加快平凉工业园区中小企业创业园、青年创业大厦、静宁工业园区电子商务产业园等特色产业示范基地建设，引进一批企业和创业团队，打造一批“双创”品牌。依托市科技孵化器，吸引优秀人才“精英创业”，支持大学生“实训创业”。加快线上线下结合、政府服务与社会服务结合的小微企业服务体系建设，提升小微企业创业创新能力。

（八）突出民生改善，大幅提升公共服务水平。进一步健全完善社会保障体系。实施全民参保登记计划，整合城乡居民医疗保险，完成医疗保险与生育保险合并，完善城乡居民大病保险制度，深化医保支付方式改革，实现省内异地就医结算。扎实推进机关事业单位养老保险制度改革。加强社会救助和社会福利体系建设，全面落实城乡低保提标政策，有效保障残疾人、妇女、未成年人和老年人权益，关爱农村留守儿童，改建市妇女儿童活动中心，建设互助老人幸福院200个，完成残疾人家庭无障碍改造100户。千方百计扩大就业。落实就业扶持政策，积极引导高校毕业生到小微企业、非公企业就业和自主创业，加强复转军人、困难家庭就业帮扶，城镇新增就业3万人，应届普通高校毕业生就业率达到85%以上。推动教育协调公平发展。进一步调整优化中心城区教育布局，统筹解决大班额问题，继续实施“改薄工程”和“温暖工程”，加快改善寄宿制学校条件，新建、改建中小学校舍9万平方米，建成标准化学校182所，办好农村小规模学校，推进义务教育均衡发展。严格落实新建小区配建幼儿园制度，鼓励社会资本建办优质民办幼儿园，实现有需求行政村全覆盖。加快发展职业教育，建成平凉职业技术学院二期。推进教育教学改革，加强素质教育，强化教师队伍建设管理，稳定农村教师队伍，提高教育教学质量。着力提升医疗服务水平。统筹推进县级公立医院综合改革，健全完善分级诊疗制度，抓好市县乡医疗卫生机构改扩建，建成市儿童专科病区，新建改建11个乡镇卫生院附属工程和周转宿舍，支持发展中医药和国医养生事业，加强基层计划生育服务管理，不断提高基本医疗和公共卫生服务能力。完善公共文化服务体系。实施文化广电体育惠民工程，建成市博物馆，开建市体育中心，加快有线广播电视网络数字化转换和双向改造，建设一批公共电子阅览室、农村数字电影固定放映点，建成乡镇社区体育中心8个、村级农民体育健身工程120个、村级文化服务中心190个。大力发展影视制作、创意动漫、广告会展等新兴文化业态。组织开展系列群众文体活动。加强非物质文化遗产传承保护，抓好历史文化名城、名镇、名村保护开发。创设和谐稳定的社会环境。启动“七五”普法，深化平安平凉建设，落实重大事项社会稳定风险评估制度，深入开展矛盾纠纷排查调处和信访突出问题化解，强化网络监管，创新社会治安综合治理机制，加快构建立体化社会治安防控体系，依法严厉打击各类违法犯罪活动，有效预防和减少不稳定因素。加大各类标准的应用考核，深入推进质量兴市。严守安全生产红线，推进监管信息化和应急救援能力建设，加大执法监管力度，杜绝重特大生产安全事故。不断改进食品药品监管方式，提升监管水平，保障人民群众饮食用药安全。完善综合防灾减灾体系，提高灾害预防和应急救助能力。依法规范宗教事务管理，支持民族乡村加快发展。全面落实双拥优抚政策，加强国防教育，促进军地军民融合发展。

各位代表，群众的幸福感就是政府工作的成就感。2016年，市政府承诺的10类34件惠民实事到年底可全部完成。2017年，我们将继续措办一批惠民实事：1.筹资3050万元，设立市农业企业扶贫互助贷款风险补偿担保基金，撬动金融资金6亿元以上，用于扶持对贫困户带动作用明显的农业产业化龙头企业和农民合作社发展。2.市县两级筹资1.9亿元，为全市639个村级互助资金协会每个配套30万元，扶持贫困对象发展产业、改善条件。3.筹措2000万元设立助保金，开展中小微企业“助保贷”业务，着力解决中小微企业融资难、融资贵问题。4.整合中心城区教育资源，建成陕西师范大学平凉附属实验中学并招生。5.开建柳树沟至崆峒山、泾川县城至田家沟、蔺家沟至莲花台3条景区道路连接线；新建村组道路200公里。6.实施泾河北部面山裸露断档区域造林绿化全覆盖工程2万亩。7.在中心城区新建盘旋路、南门什字人行过街设施，建成公共停车场10处，新增停车位1000个以上。8.筹资5亿元，实施中心城区和泾川、静宁两县县城天然气管道项目，实现居民供气5万户。9.实施中心城区和六县县城棚户区改造8801户；完成农村易地搬迁2020户7858人；改造农村危房7002户。10.实施农村饮水安全巩固提升工程13处，解决10万人饮水不稳定问题。

加强政府自身建设

面对艰巨繁重的改革发展任务，我们将自觉增强政治意识、大局意识、核心意识和看齐意识，认真践行“三严三实”，深入开展“两学一做”学习教育，全面履职尽责，勇担发展重任，努力建设组织放心信任、人民群众满意信赖的政府。

强化理论武装，深入推进学习型政府建设。认真学习领会习近平总书记系列重要讲话精神和党中央治国理政的新理念、新思想、新战略，不断增强贯彻落实党的路线方针政策的自觉性和坚定性。深入研究国家宏观政策、行业法规政策和产业政策，找准落实落地的切入点和结合点，不断提高把握政策、谋划工作、推动发展的能力。健全完善政府工作人员教育培训长效机制，有针对性地开展能力素质培训，增强专业素养和业务能力，不断提升解决实际问题的水平。

严格依法行政，深入推进法治政府建设。坚持依法科学民主决策，严格按照法定权限和程序行使权力、履行职责，自觉接受监督，认真办理人大代表建议和政协委员提案。加快综合行政执法改革，推进县区及以下市场监管体制改革，调整理顺城市街道社区管理体制。加快推进电子政务建设，大力推广“互联网+政务”，全面推行网上审批和并联审批，扩大行政权力运行、公共资源交易等领域信息公开，完善政府新闻发布制度，提高政府工作透明度。

勇于担当实干，深入推进责任政府建设。始终把敢于担当作为全体政府工作人员的自觉行动，切实强化使命担当、责任担当，勇于攻坚克难、动真碰硬、善作善成，创造出经得起历史和人民检验的工作实绩。加大常态化督查力度，下功夫整治重布置、轻落实，有令不行、有禁不止，推诿扯皮、敷衍塞责等问题，树立说了就算、定了就干、干就干好的良好政风，确保各项工作高标准推进、高质量完成。

坚持履职为民，深入推进服务型政府建设。进一步畅通民意表达渠道，及时回应社会关切，确保事事有回音、件件有结果。坚持重心下沉、服务下移，把工作着力点更多放到基层和生产一线，用工作实绩赢得群众的信任和支持。更加注重发挥属地化服务管理职能，为企事业单位和各类市场主体搞好服务，调动共建共享积极性，共同提升平凉整体形象。

坚持勤政廉政，深入推进廉洁政府建设。认真学习贯彻党的十八届六中全会精神，始终把纪律和规矩挺在前面，全面落实 “3783”主体责任和 “866”衡量标准，严格执行中央八项规定和省委“双十条”、市委“十二条”规定，强化对权力运行的制约和监督，对财政资金分配使用、国有资产经营、政府投资、政府采购、土地征用招拍挂、建筑市场管理等重点领域和部门，加强常态化监管、随时性提醒、及时性规范，坚决纠正损害群众利益的不正之风，严肃查处“四风”和腐败问题，树立清正廉洁的良好形象。

各位代表！困难和挑战考验着我们，责任和使命激励着我们。让我们在市委的坚强领导下，凝聚起全市人民的智慧和力量，开拓创新，砥砺奋进，为建设幸福活力美好平凉而努力奋斗！
